# A Fe-incorporated bioreactor platform exhibiting antimalarial activity and enhanced response to artemisinin

**DOI:** 10.1128/aac.01390-25

**Published:** 2026-02-20

**Authors:** Yinyue Li, Shuang Li, Huajun Li, Yun Yang, Jiahui Xu, Ying Deng, Xinlong He, Fang Tian, Jin-Hee Han, Eun-Taek Han, Juqun Xi, Feng Lu

**Affiliations:** 1 School of Basic Medical Sciences, Faculty of Medicine, Yangzhou University 38043, Yangzhou, China; 2 Autobio Diagnostics Co., Ltd., Zhengzhou, China; 3 School of Traditional Chinese Medicine, Faculty of Medicine, Yangzhou University 38043, Yangzhou, Jiangsu, China; 4 Key Laboratory of the Jiangsu Higher Education Institutions for Integrated Traditional Chinese and Western Medicine in Senile Diseases Control (Yangzhou University), Yangzhou, Jiangsu, China; 5 Department of Tropical Medicine, School of Medicine, Kangwon National University 85082, Chuncheon, Korea; 6 The Key Laboratory of the Jiangsu Higher Education Institutions for Nucleic Acid & Cell Fate Regulation, Yangzhou University 38043, Yangzhou, China; 7 Affiliated Hospital of Yangzhou University, Yangzhou University 38043, Yangzhou, China; The Children's Hospital of Philadelphia, Philadelphia, Pennsylvania, USA

**Keywords:** malaria, Fe^2+^, PDA@Fe/P, artemisinin (ART), ART-resistant malaria parasites

## Abstract

While malaria parasites rely on labile Fe^2+^ pools for survival, excess Fe^2+^ acts as a Fenton reagent, inducing cytotoxicity via reactive oxygen species and membrane disruption, highlighting iron homeostasis as a key therapeutic vulnerability. To test the feasibility of iron ions in inhibiting *Plasmodium* parasites, we developed Fe^2+^-loaded polydopamine nanoparticles (PDA@Fe/P) that exploit the parasite’s iron-dependent vulnerabilities through dual mechanisms: (i) sustained Fe^2+^ release triggers Fenton reactions, generating cytotoxic hydroxyl radicals that overwhelm antioxidant defenses, and (ii) restoration of artemisinin (ART) activation in resistant parasites by supplementing the diminished Fe^2+^ pool. *In vitro* testing against five *P. falciparum* strains (including chloroquine- and ART-resistant variants) demonstrated potent, antimalarial activity, with efficacy 20-fold higher than free FeCl_2_ due to enhanced solubility and controlled release. While *in vivo* studies in *P. berghei*-infected mice showed transient parasite suppression without toxicity, the relatively high IC_50_ precludes standalone use. Crucially, PDA@Fe/P enhanced the activity of ART against *P. falciparum* strain with partial ART resistance conferred by Kelch13 mutation, by counteracting mutation-induced impairments in hemoglobin endocytosis and heme bioavailability—key determinants of ART activation. Analysis of lipid peroxidation levels revealed that Fe^2+^ delivered via PDA@Fe/P amplifies oxidative stress responses in resistant parasites, indicating its ability to enhance the sensitivity of ART-resistant strains to ART. Our findings establish iron-based delivery strategies as a promising approach to potentiate existing antimalarials and combat resistance through the targeted disruption of redox homeostasis.

## INTRODUCTION

Malaria, a mosquito-borne disease caused by *Plasmodium* parasites, is one of the deadliest parasitic diseases worldwide, resulting in over 600,000 fatalities each year (1). Currently, no highly effective malaria vaccine exists, and chemotherapy remains a cornerstone of malaria control. Artemisinin-based combination therapies (ACTs) remain the last line of defense; however, drug resistance—an inevitable challenge for all antimalarial drugs—has emerged, including resistance to artemisinin (ART) in the most lethal species, *Plasmodium falciparum* (2, 3). This underscores an urgent need for novel compounds and drug combinations to mitigate the looming shortage of viable therapeutic options.

Iron is an essential metal for all living organisms, including malaria parasites, which require it for growth and replication. As a eukaryote, *P. falciparum* relies on labile iron pools to synthesize essential protein cofactors, such as iron-sulfur (Fe-S) clusters and di-iron centers, which play critical roles in fundamental metabolic pathways, including mitochondrial electron transport and DNA replication/repair machinery (4, 5). It has been indicated that providing iron supplements in the active form (Fe^2+^) may increase the bioavailability of active iron in the labile pool, facilitating the rapid growth of parasites (6). However, excess intracellular iron can be toxic (7, 8): Fe^2+^ acts as a Fenton reagent, triggering membrane disruption and other cytotoxic effects (9–12). These mechanistic insights highlight the disruption of iron homeostasis as a promising therapeutic strategy against malaria (13).

ART undergoes Fe^2+^-mediated reduction within heme, generating reactive radicals that induce protein alkylation (14). In contrast, *P. falciparum* strains carrying Kelch13 mutations exhibit partial ART resistance, clinically manifested as delayed parasite clearance during treatment. This resistance involves an adaptive response that reduces intracellular heme bioavailability, thereby lowering oxidative stress and limiting the Fe^2+^ pool required for ART activation via endoperoxide cleavage (3). Consequently, pharmacological strategies aimed at restoring the Fe^2+^-dependent activation pathway—particularly by counteracting hemoglobin digestion-mediated iron depletion—represent a promising approach to reverse ART partial resistance in *P. falciparum*.

Recent advancements in nanotechnology have demonstrated its potential as a powerful tool for developing novel antimalarial agents, offering distinct advantages such as enhanced drug bioavailability and stability, reduced toxicity, and targeted drug delivery (15, 16). Notably, metal nanoparticles (NPs) synthesized via nanotechnology have shown promising antimalarial activity in therapeutic applications (17). Polydopamine (PDA) materials have garnered significant interest due to their exceptional metal-ion chelation capacity, antioxidant properties, biocompatibility, and photothermal conversion efficiency (18–20). Inspired by these properties, we synthesized polydopamine nanoparticles (PDAs) as photothermal agents and coated them with iron phosphate shells (PDA@Fe/P) (21, 22). Herein, we explored the dual antimalarial capabilities of PDA@Fe/P, which manifest in two key aspects: (i) inducing intracellular Fe^2+^ overload, which may lead to parasite death through mechanisms possibly involving oxidative stress, such as reactive oxygen species (ROS) accumulation and changes in mitochondrial membrane potential; (ii) significantly enhancing the efficacy of dihydroartemisinin (DHA) against ART-resistant *P. falciparum* strains by promoting ART activation. This synergistic mechanism resulted in potent growth inhibition of both drug-sensitive and resistant malaria parasites.

## RESULTS

### Preparation and characterization of PDA@Fe/P

**Fig 1:**
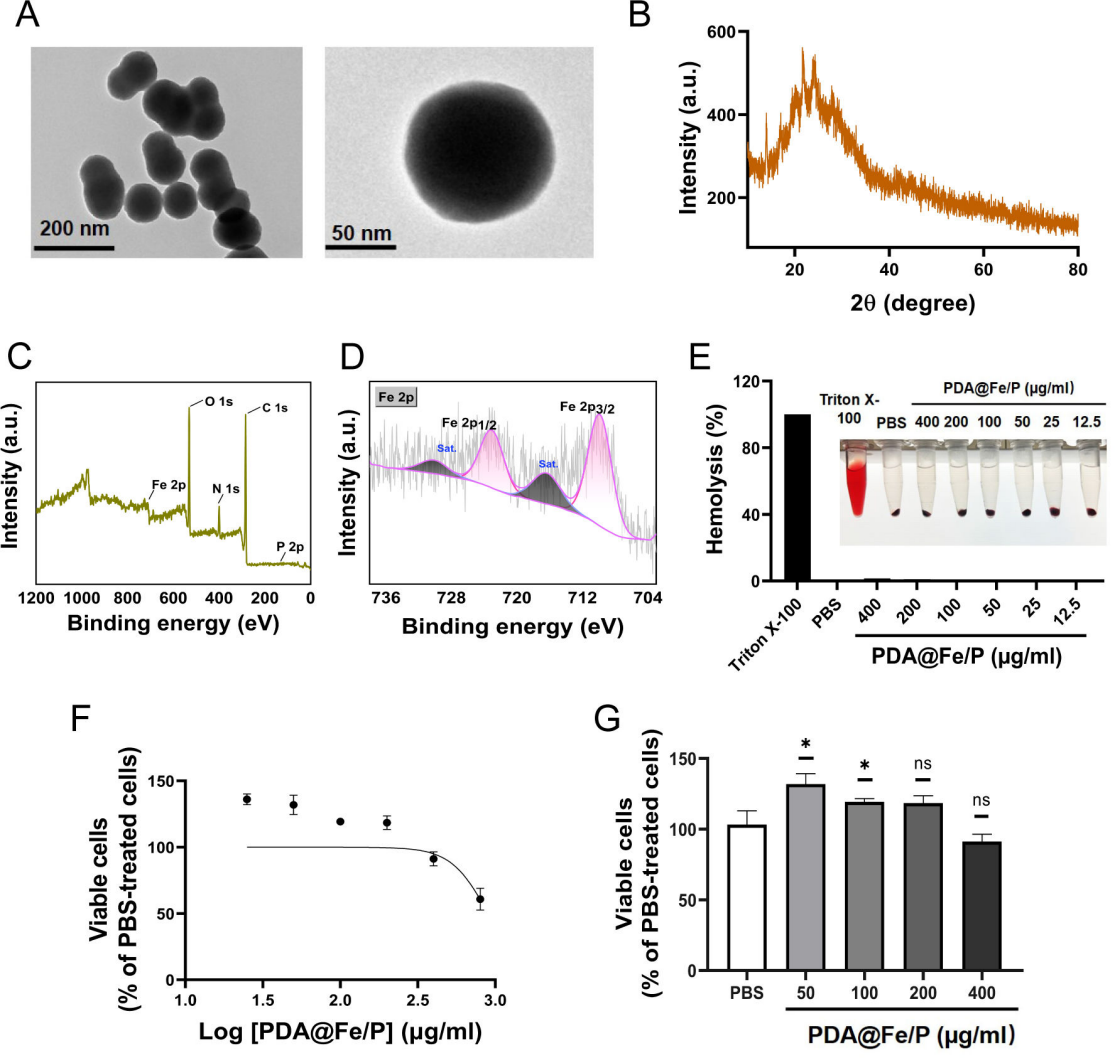
Synthesis and characterization of PDA@Fe/P. (**A**) TEM images of PDA@Fe/P. (**B**) XRD analysis of PDA@Fe/P. (**C**) XPS spectrum of PDA@Fe/P: survey spectrum. (**D**) High-resolution XPS spectrum of Fe 2p orbitals. (**E**) Hemolysis assessment of PDA@Fe/P at varying PDA@Fe/P concentrations (*n* = 3). (**F**) and (**G**) Cell viability of HUVECs following 24 h exposure to different concentrations of PDA@Fe/P, measured by MTT assay (*n* = 3). *P < 0.05 and **P < 0.01 compared with the blank control group. ns, not significant (*P* ≥ 0.05).

To verify the successful synthesis of PDA@Fe/P and elucidate its structural features, we first performed a series of material characterization experiments, including transmission electron microscopy (TEM), X-ray diffraction (XRD), and X-ray photoelectron spectroscopy (XPS), to analyze morphology, crystallinity, and elemental composition. The PDA@Fe/P was synthesized via a simple one-step method (22, 23). TEM images (Fig. 1A) revealed that the as-synthesized PDA@Fe/P exhibited uniform spherical morphology with a diameter of 100 nm. To further confirm the successful synthesis of PDA@Fe/P, XRD and XPS measurements were conducted. The XRD results in Fig. 1B demonstrated the amorphous nature of the PDA@Fe/P, confirming the successful preparation of PDA@Fe/P, while the full XPS survey spectrum exhibited characteristic peaks corresponding to C 1s, N 1s, O 1s, P 2p, and Fe 2p, with atomic percentages of 33.54%, 7.54%, 33.85%, 1.08%, and 23.99%, respectively (Fig. 1C; Fig. S1A). The high-resolution spectrum of the Fe 2p orbital revealed peaks of Fe 2p1/2 and Fe 2p3/2 at 723.8 and 709.88 eV, indicating that the chemical state of Fe in PDA@Fe/P was primarily in the +2 valence state (Fig. 1D) (24). Therefore, the measured Fe atomic percentage represents the surface Fe species originating from FeCl_2_ incorporated into the PDA/P matrix, rather than the exact proportion of FeCl_2_ remaining in the final product. To directly evaluate Fe^2+^ release from PDA@Fe/P, the material was incubated in culture medium at 37°C, and Fe^2+^ concentrations were quantified at multiple time points using the probe-based method. The results demonstrated a sustained release pattern throughout the observation period (Fig. S1E), indicating that PDA@Fe/P is capable of continuously liberating iron ions, which may underlie its biological activity.

To assess the *in vitro* biocompatibility of PDA@Fe/P and exclude the possibility that its antimalarial activity is mediated via hemolysis, we conducted hemolysis tests using rabbit erythrocytes and cytotoxicity assays on human umbilical vein endothelial cells (HUVECs). To evaluate the biocompatibility and potential *in vivo* toxicity of PDA@Fe/P, we performed systematic hemolysis and cytotoxicity assays. To determine whether the antimalarial mechanism involved erythrocyte lysis, hemolytic activity was quantified using defibrinated rabbit erythrocytes, with PBS (negative control), 0.4% Triton X-100 (positive control), and chloroquine (CQ, reference drug) as controls. The results revealed that both PDA@Fe/P and CQ exhibited negligible hemolysis (<5% at IC_50_ concentrations) with no detectable erythrocyte membrane disruption (Fig. 1E; Fig. S2A). In stark contrast, the precursor FeCl_2_·4H_2_O showed concentration-dependent hemolysis, inducing significant RBC lysis at high concentrations (27.8% at 7,544.9 μg/mL; 28.8% at 15,089.8 μg/mL) while remaining non-hemolytic at lower doses (Fig. S2A).

To investigate the cytotoxic effects of PDA@Fe/P on HUVECs, cell suspensions were seeded in 96-well plates and treated with PDA@Fe/P after adherence, followed by CCK-8 assay analysis. Our results indicated that PDA@Fe/P exhibited no significant cytotoxicity in the concentration range of 50–400 μg/mL, as cell viability remained comparable to that of the blank control (Fig. 1F and G). Notably, a mild but statistically significant proliferative effect was observed at 50 μg/mL. Higher concentrations (100–400 μg/mL) showed no marked changes in cell viability compared with the control. In parallel experiments with FeCl_2_·4H_2_O (Fig. S2B and C), notable cytotoxicity only occurred at very high concentrations (>3,772.45 µg/mL), with no significant effects observed at <7,544.9 µg/mL. The 15,089.8 μg/mL treatment group showed highly significant differences compared to controls. These findings reveal that PDA@Fe/P exhibits significantly enhanced bioactivity at concentrations orders of magnitude lower than FeCl_2_·4H_2_O, strongly suggesting that the unique structural and chemical properties of the PDA@Fe/P composite play a pivotal role in amplifying its biological efficacy.

### PDA@Fe/P exhibits broad-spectrum antimalarial activity

We next systematically compared the parasite inhibition efficiency of PDA@Fe/P with that of other iron-based antimalarial nanomaterials and reference drugs, including CQ and FeCl_2_·4H_2_O, against multiple *Plasmodium falciparum* strains, to evaluate its activity spectrum and relative potency. Meanwhile, the PDA raw material was included as a negative control in the experimental system. As shown in Table S1, at a concentration of 100 μg/mL, PDA@Fe/P demonstrated significantly higher parasite inhibition (60.0%) compared to other iron-based nanomaterials, including PDA@Fe(III)P, Fe-doped carbon spheres (Fe-C), and Fe/N-doped carbon spheres (Fe-N) (25). PDA@Fe(III)P showed moderate inhibition (43.1%), while Fe-C and Fe-N demonstrated minimal activity (3.6% and 7.0%, respectively). The raw PDA material itself exhibited only limited activity, with an inhibition rate of 18.5%. Based on these results, PDA@Fe/P was selected for further investigation.

**Fig 2:**
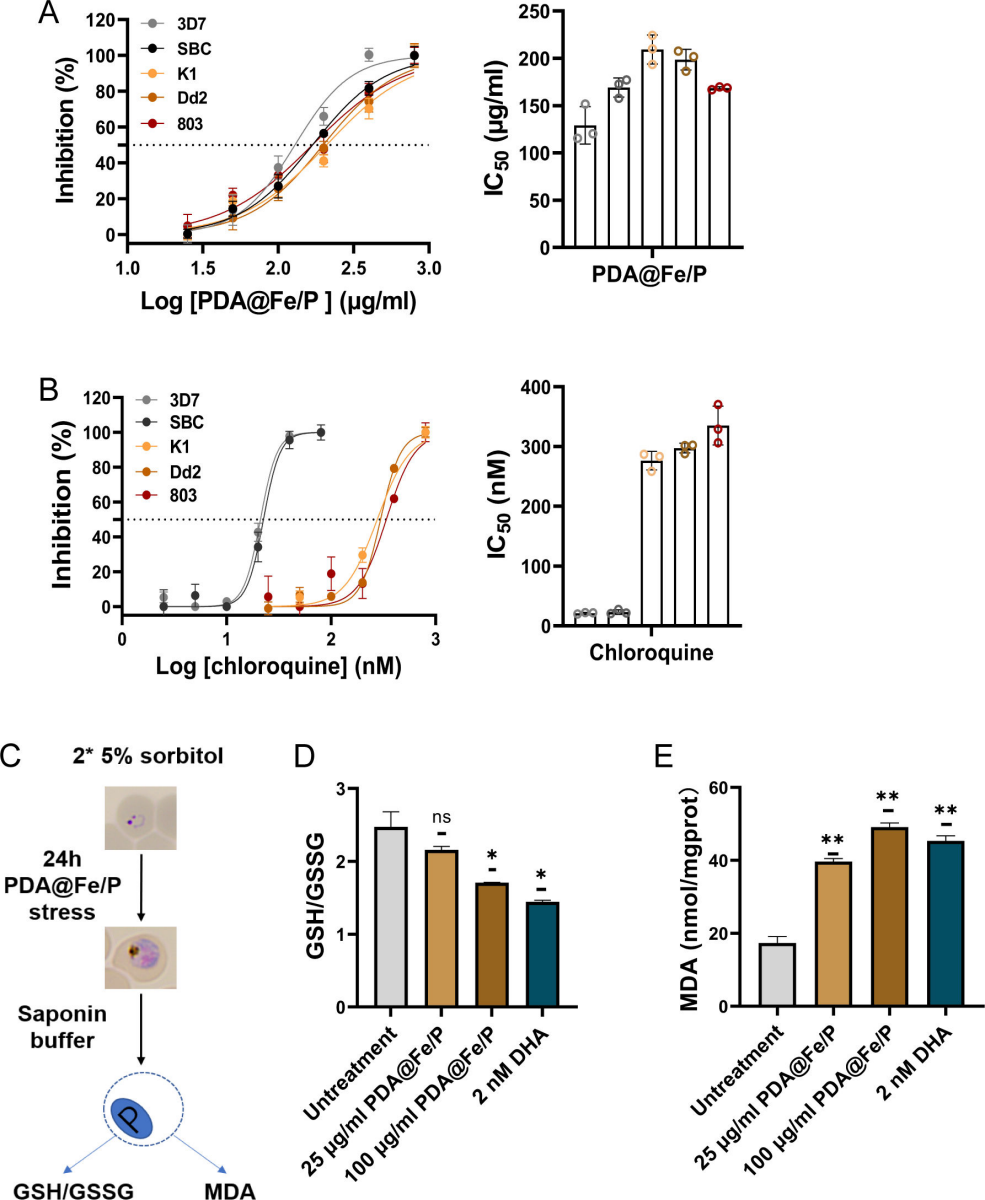
Preliminary screening of *in vitro* antimalarial activity of PDA@Fe/P. (**A**) and (**B**) IC_50_s of PDA@Fe/P against *P. falciparum* strain. Data are individual replicates or means ± SD of 10,000 red blood cells (RBCs) from two independent assays with three technical replicates. (**C**) Schematic illustration of the experimental design. A representative image has been used to illustrate the parasite development stages. (**D**) Effect of PDA@Fe/P on intracellular GSH/GSSG ratio in infected red blood cells (iRBCs). (**E**) Effect of PDA@Fe/P on malondialdehyde (MDA) levels in RBCs. ns, not significant (*P* ≥ 0.05).

The *in vitro* antimalarial activity of compounds CQ, PDA@Fe/P, and FeCl_2_·4H_2_O was evaluated against four *P. falciparum* (3D7, 803, Dd2, and K1) and a field isolate (SBC). Among these, 3D7 and SBC were identified as CQ-sensitive, with IC_50_ values of 21.08 and 22.54 nM, respectively, whereas strains K1, Dd2, and 803 were CQ-resistant, exhibiting significantly higher IC_50_ values of 276.4, 297.3, and 337.3 nM, respectively (Fig. 2A and B). Both PDA@Fe/P and FeCl_2_·4H_2_O demonstrated potent antimalarial effects against all tested strains, including CQ-resistant isolates. PDA@Fe/P exhibited IC_50_ values of 129.1, 168.8, 209.3, 198.6, and 168.4 μg/mL against 3D7, SBC, K1, Dd2, and 803, respectively (Fig. 2A and B), while FeCl_2_·4H_2_O showed higher IC_50_ values of 2,330, 3,995, 4,668, 3,798, and 4,015 μg/mL against 3D7, SBC, K1, Dd2, and 803, respectively (Table S2). These results indicate that PDA@Fe/P possesses significantly stronger antimalarial activity than FeCl_2_·4H_2_O and maintains potent efficacy against both CQ- and ART-resistant *P. falciparum* strains, suggesting its promising potential as a novel antimalarial agent.

### PDA@Fe/P interferes with the redox homeostasis of parasites

The biological pathways governing iron homeostasis are highly complex, with Fe^2+^-induced Fenton reactions being implicated in diverse pathological processes. During the intraerythrocytic developmental cycle (IDC), malaria parasites exhibit a high metabolic demand, consuming substantial amounts of glucose for energy production and degrading large quantities of hemoglobin to support their growth and proliferation (26). Heme represents a toxic byproduct of this process, which the parasite subsequently converts into hemozoin—an inert biocrystalline material containing oxidized heme (Fe^3+^). Although this detoxification pathway mitigates heme toxicity, current evidence suggests that Fe^2+^ concentrations in infected RBCs (iRBCs) remain significantly elevated compared to those in serum and healthy tissues (26). The resulting oxidative damage creates a highly pro-oxidant intracellular environment that challenges parasite survival (27).

The parasite’s antioxidant defense system is primarily composed of glutathione (GSH) and a suite of thioredoxin-dependent proteins, which collectively constitute a crucial redox regulatory network for maintaining cellular homeostasis and counteracting oxidative stress (28, 29). Malondialdehyde (MDA), a key end product of lipid peroxidation, serves as an important biomarker for oxidative damage to cell membranes. To investigate the possible mechanism of action of PDA@Fe/P, oxidative stress markers in the parasites were measured, including MDA level and GSH/GSSG, to assess whether PDA@Fe/P disrupts redox homeostasis. Parasites, after two successive synchronizations with 5% sorbitol to obtain tightly synchronized ring-stage cultures, were treated with PDA@Fe/P for 24 h. They were subsequently obtained by saponin-lysing infected red blood cells (RBCs) and subjected to the oxidative stress assays (Fig. 2C). As shown in Fig. 2D and E, as anticipated, with increasing concentrations of PDA@Fe/P treatment, the MDA content increased significantly, while the GSH/GSSG ratio exhibited an opposite trend. These results indicate that PDA@Fe/P causes oxidative damage to the parasites by disrupting their redox homeostasis. Consistent with earlier studies showing the excellent blood compatibility of PDA, treatment with PDA@Fe/P led to a significant increase in the GSH/GSSG ratio in RBCs relative to the untreated control (Fig. 2D). This finding suggests that PDA@Fe/P enhances the antioxidant capacity and maintains redox homeostasis in RBCs, demonstrating good biocompatibility with host cells.

### PDA@Fe/P exerts an inhibitory effect on *P. berghei in vivo*

**Fig 3:**
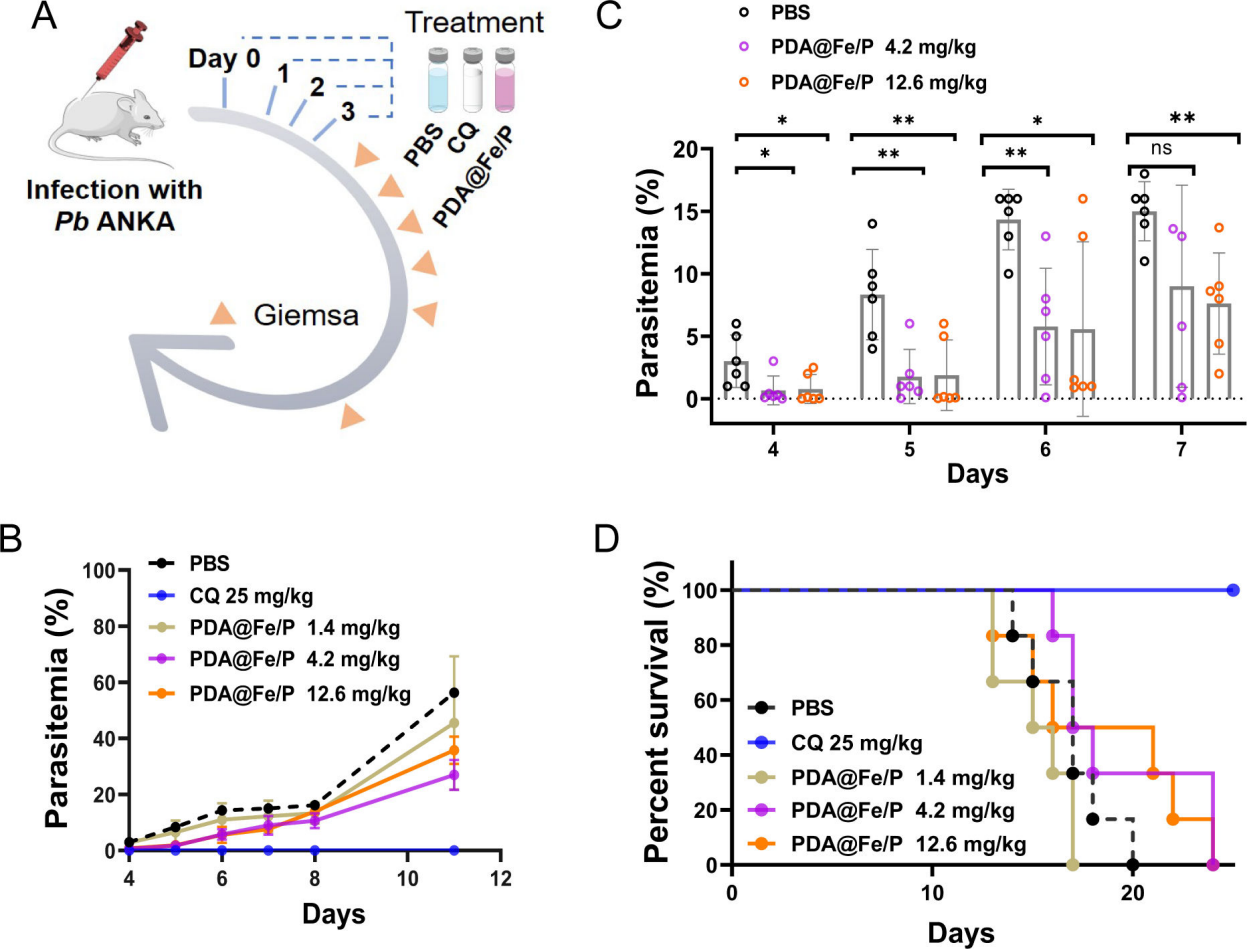
Antimalarial assessment of PDA@Fe/P *in vivo*. (**A**) Experimental schedule for the 4-day suppressive test, indicating treatment initiation relative to parasite inoculation and observation periods and (**B**) Kaplan-Meier survival curves of *P. berghei*-infected female BALB/c mice (*n* = 6 per group) receiving PBS control, PDA@Fe/P at various doses, or standard antimalarial drug treatment. (**C**) and (**D**) Inhibitory activity of PDA@Fe/P at doses of 1.4, 4.2, and 12.6 mg/kg against *Pb in vivo*. Data are means ± SD from two independent assays with six mice each time. Statistical analyses between the two groups were done by Student’s *t*-tests, **, *P* < 0.01, *, *P* < 0.05; ns, not significant (*P* ≥ 0.05).

The *in vivo* antimalarial effect of PDA@Fe/P was evaluated through a standard 4-day suppressive test in BALB/c mice infected with *P. berghei* (Fig. 3A). The growth of *P. berghei* and the survival of mice were observed after four consecutive days of administration. The results showed that PDA@Fe/P had a significant antimalarial effect in mice. Microscopic evaluation of parasitemia progression revealed significant antimalarial activity of PDA@Fe/P (Fig. 3B and C). On day 4 post-infection, the PBS control group showed 3% parasitemia, while PDA@Fe/P treatment groups exhibited markedly lower parasitemia (0.67% at 4.2 mg/kg and 0.77% at 12.6 mg/kg), representing 77.3–77.7% inhibition relative to controls (Fig. 3C). The differences in parasitemia became more pronounced by day 6, with PBS controls reaching 14.3% infection compared to 5.8% (4.2 mg/kg) and 5.6% (12.6 mg/kg) in treated groups (59.4–60.8% inhibition). By day 7, when PBS controls plateaued at 15% parasitemia, PDA@Fe/P-treated mice maintained significantly lower parasitemia (9% at 4.2 mg/kg and 7.6% at 12.6 mg/kg), demonstrating 40–49.3% inhibition (Fig. 3C). Notably, the 1.4 mg/kg group showed no statistically significant difference from PBS controls, indicating a concentration-dependent therapeutic effect. These findings establish that PDA@Fe/P significantly suppresses malaria proliferation in a dose-responsive manner while maintaining effect throughout the critical 7-day observation period. No parasites were detected in the CQ-treated group throughout the observation period. The inhibitory effect of PDA@Fe/P was calculated as the ratio of its infection rate to that of the PBS group. PDA@Fe/P exhibited strong antimalarial activity, but its effect declined by day 7.

Kaplan-Meier survival curves comparing the PDA@Fe/P treatment group with the PBS control group showed no statistically significant difference in survival, as assessed by the log-rank test (*P* > 0.05) (Fig. 3D). However, a closer examination of the survival data reveals a dose-dependent extension in the PDA@Fe/P-treated groups. All mice in the PBS control group died by day 20 after infection. In contrast, treatment with PDA@Fe/P demonstrated a dose-dependent extension of survival, with the last remaining animals in both the 4.2 and 12.6 mg/kg treatment groups succumbing on day 25, representing a 25% prolongation of survival compared to controls (Fig. 3D). In contrast, no mortality was observed in the CQ-treated group throughout the entire study period.

### Co-treatment of ART-resistant parasites with ART and PDA@Fe/P synergistically reduces parasite survival

**Fig 4:**
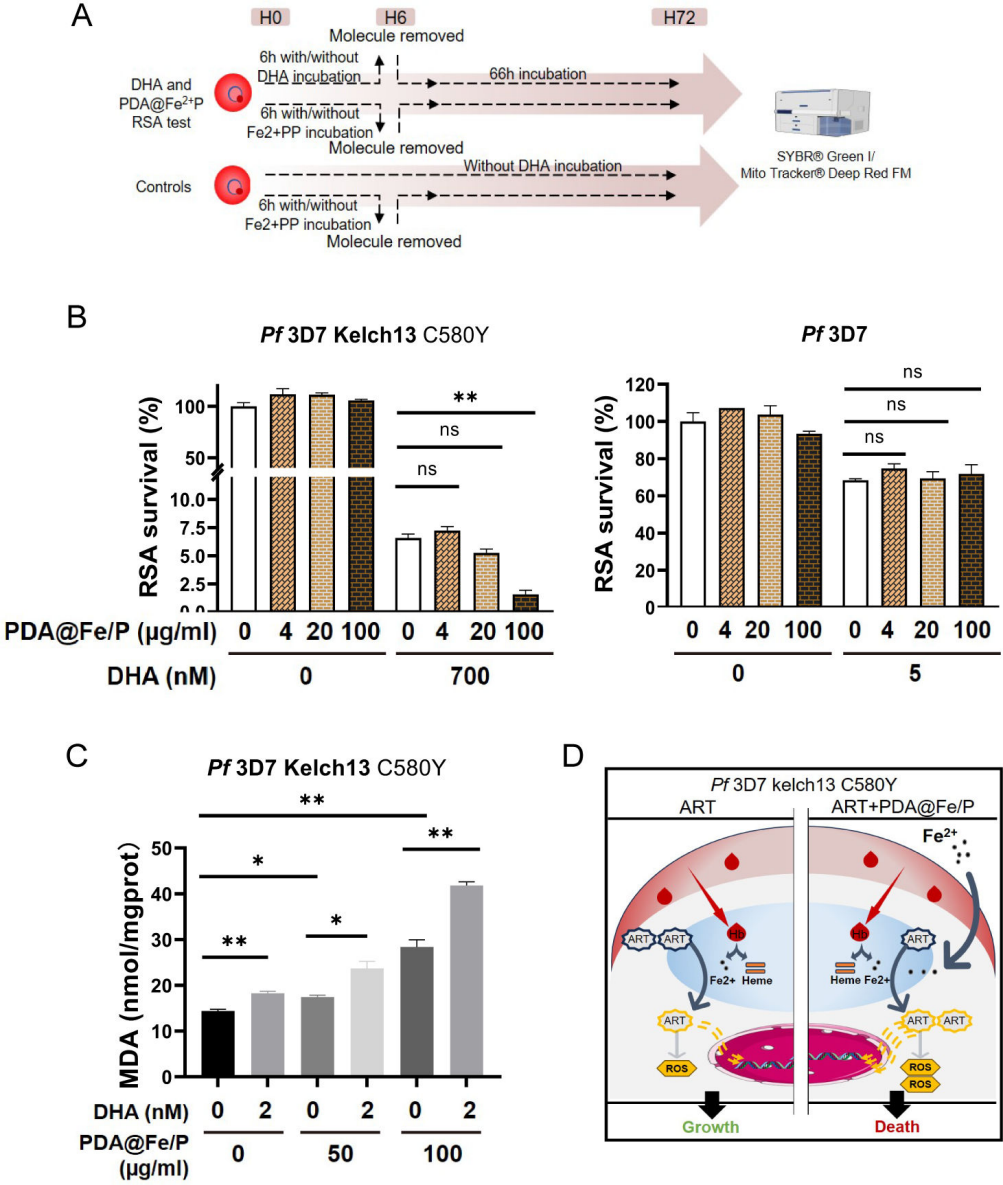
Co-treatment with ART and PDA@Fe/P synergistically reduces the survival of artemisinin-resistant parasites. (**A**) RSA tests template. (**B**) RSA of PDA@Fe/P and DHA against *P. falciparum* strains, including the ART-sensitive 3D7 and ART-resistant Kelch13^C580Y^ strain. (**C**) Measurement of MDA levels as an indicator of lipid peroxidation in *P. falciparum* strain partial resistant to ART following co-treatment with PDA@Fe/P and ART. MDA production was quantified using a thiobarbituric acid reactive substances (TBARS) assay, revealing significantly higher lipid peroxidation in the co-treatment group compared with ART alone. (**D**) Proposed mechanism illustrating how PDA@Fe/P replenishes the labile iron pool to restore ART activation and enhance oxidative damage, thereby overcoming ART resistance.*, *P* < 0.05; **, *P* < 0.01, ns, denotes not significant (*P* ≥ 0.05).

Resistance against the frontline antimalarial drug ART is a major threat to malaria control and elimination efforts. Parasites develop resistance by reducing hemoglobin uptake and digestion, which lowers oxidative-stress levels and prevents the activation of ART (3). Given that ART partial resistance manifests through reduced ring-stage susceptibility (allowing parasites to withstand the drug’s short pharmacokinetic exposure), we systematically evaluated PDA@Fe/P’s ability to resensitize resistant parasites using standardized ring-stage survival assays (RSAs) (Fig. 4A). RSAs with *P. falciparum* ring stages co-treated with PDA@Fe/P and DHA, one of the ART derivatives used as first-line antimalarials, showed synergistically reduced survival of Kelch13^C580Y^ at 100 μg/mL PDA@Fe/P (Fig. 4B). As a comparison, FeCl_2_ also exhibited synergistic activity with DHA in RSA assays with the 3D7 Kelch13 C580Y strain; however, its toxicity was pronounced at higher concentrations. Notably, FeCl_2_ alone showed measurable parasiticidal activity, further indicating its intrinsic antimalarial potential (Fig. S3). In contrast, PDA@Fe/P exhibited no synergistic activity with low-concentration DHA against the ART-sensitive 3D7 strain (Fig. 4B). These findings suggest that PDA@Fe/P specifically potentiates ART activation in resistant parasites, potentially compensating for reduced drug activation caused by impaired hemoglobin endocytosis in resistant strains.

To evaluate the oxidative stress levels, the final product of lipid peroxidation was measured using a micro-MDA assay reagent kit. Compared with the untreated control, PDA@Fe/P could enhance the lipid peroxidation level caused by ART in Kelch13^C580Y^ mutant (Fig. 4C and D). Altogether, PDA@Fe/P significantly enhanced the antimalarial efficacy of ART by inducing oxidative stress (as evidenced by increased MDA levels) in ART-resistant parasites through potentiating ART activation. Taken together, PDA@Fe/P could thus both reduce the survival of ART-resistant parasites when combined with ART and independently inhibit the growth of ART-resistant *P. falciparum*.

## DISCUSSION

The increasing failure of current malaria control strategies is primarily attributed to the emergence and rapid spread of drug-resistant parasite strains (30). This underscores an urgent need for new antimalarial drugs with innovative modes of action.

Malaria parasites’ obligate intraerythrocytic development creates a critical metabolic dependency on host hemoglobin catabolism, generating substantial quantities of redox-active Fe^2+^ and toxic heme as byproducts (31–33). While *P. falciparum* detoxifies most heme through biomineralization into inert hemozoin crystals, its evolutionary loss of functional heme oxygenase activity represents a significant metabolic vulnerability. This vulnerability is pharmacologically exploitable: for example, deferoxamine exhibits potent antimalarial activity by selectively chelating labile Fe^2+^ pools (19, 34, 35), triggering iron dysregulation that initiates a cascade of cytotoxic events. Specifically, Fe^2+^-mediated Fenton reactions generate hydroxyl radicals, inducing membrane-destabilizing lipid peroxidation and ultimately compromising parasite viability (9–11, 36).

To counteract such oxidative threats, malaria parasites have evolved a sophisticated iron regulatory network. Intriguingly, while the iron-dependent cell death pathway ferroptosis remains mechanistically undefined in malaria parasites, multiple lines of evidence suggest its involvement in antimalarial drug action (37). Both ART and CQ induce characteristic ferroptosis markers in malaria parasites, and pharmacological inhibition of ferroptosis attenuates ART-mediated parasite killing (38, 39). These findings collectively establish iron homeostasis disruption as a compelling antimalarial strategy, with several organometallic compounds currently under clinical evaluation (13). However, a library of ~150 ferrocene-conjugated analogs (or metallocene derivatives) based on known antimalarials showed largely disappointing *in vitro* activity compared to their organic parent compounds (13). Notably, supplementing iron in its active form may enhance the bioavailability of labile iron, thereby facilitating the rapid growth of parasites (6).

Among these, ferroquine (FQ) stands out as one of the few effective derivatives, where strategic incorporation of the ferrocenyl moiety into CQ’s side chain avoids steric hindrance to the drug’s pharmacophore (40).

Building on these insights, we synthesized PDA@Fe/P via a one-pot method: nanospheres with Fe^2+^ at their core, featuring a 100 nm particle size. *In vitro* studies demonstrated potent antimalarial activity of PDA@Fe/P against five *P. falciparum* strains (3D7, SBC, Dd2, K1, and 803), with IC_50_ values (129.1–209.3 μg/mL), approximately 20-fold lower than the precursor FeCl_2_·4H_2_O (2,330–4,668 μg/mL). This dramatic enhancement likely stems from the NPs’ superior solubility, dispersibility, and sustained Fe^2+^ release (41). Notably, PDA@Fe/P showed consistent efficacy across CQ-sensitive (3D7 and SBC), CQ-resistant (Dd2 and K1), and ART/CQ-dual-resistant (803) strains (<2-fold variation), confirming its broad-spectrum potential against drug-resistant malaria parasites. *In vivo* evaluation via the Peter’s test revealed that PDA@Fe/P significantly reduced *P. berghei* parasitemia (days 4–7) without extending survival time or causing toxicity, though its transient effect and relatively high IC_50_ preclude direct therapeutic use. Notably, these findings nonetheless validate the efficacy of carrier-based therapeutic strategies that utilize Fe^2+^ as a key effector. By targeting the homeostasis of dynamic Fe^2+^ pools, Fe^2+^ can disrupt parasite iron metabolism and trigger cytotoxic cascades, which underscores the need for further optimization to enhance its efficacy and duration of action.

The oxidative damage resulting from extensive hemoglobin degradation subjects the parasites to a state of high oxidative stress. To counter this, malaria parasites' antioxidant defense system relies primarily on GSH and a range of thioredoxin-dependent proteins (28). As the most abundant low-molecular-weight redox-active thiol in parasites, GSH neutralizes oxidative byproducts and maintains an intracellular reducing environment (29); notably, fluctuations in GSH levels may also affect ART sensitivity (42). In the present study, we simultaneously detected MDA and the GSH/GSSG ratio in parasites treated with PDA@Fe/P. Results showed that MDA levels increased significantly with PDA@Fe/P concentration, whereas GSH levels trended downward, suggesting that PDA@Fe/P induces oxidative damage by disrupting redox homeostasis. Consistent with earlier studies reporting the excellent blood compatibility of PDA, we found that PDA@Fe/P treatment led to a significant increase in the GSH/GSSG ratio in RBCs compared with the untreated control. This suggests that PDA@Fe/P enhances the antioxidant capacity and maintains redox homeostasis in RBCs, thereby demonstrating good biocompatibility with host cells while selectively inducing oxidative stress in the parasites.

ART resistance, a major threat to malaria control, emerged in *P. falciparum* strains in Southeast Asia and has now been detected in Africa (2, 43), highlighting the urgent need for counterstrategies (44, 45). Resistance is primarily linked to adaptive changes that disrupt ART’s iron-dependent activation mechanism, through which ART relies on iron-mediated lactone ring opening to fully expose its endoperoxyl functional group, a process essential for its rapid activation (14), and mirroring its selective toxicity in iron-rich cancer cells (46), ART’s activity against *Plasmodium* is strictly iron-dependent, with its efficacy in infected RBCs directly correlating with iron levels (47). In resistant parasites, however, this activation pathway is impaired through reduced hemoglobin endocytosis, an adaptive response that both lowers oxidative stress and limits the Fe^2+^ pool available for ART activation via endoperoxide cleavage (3). This limitation can be addressed by slow release of the catalyst Fe^2+^ within the cells, thereby sustainably activating ART. Herein, we have considered the approach where surface-coated Fe^2+^ slowly provides a gradual release, leading to persistent ART activation, enabling the generation of radical species via endoperoxide cleavage. Experiments demonstrated that combining ART with PDA@Fe/P synergistically reduced the survival of ART-resistant parasites, indicating that PDA@Fe/P could be crucial in restoring ART efficacy against resistant strains.

In *P. falciparum*, iron-dependent ART exerts its activity through oxidative stress by damaging cellular macromolecules such as proteins and DNA (48–50). In this study, we observed that the combination of PDA@Fe/P and ART generated greater oxidative stress in the presence of an iron catalyst compared to ART alone. Engineered metal NPs provide distinct pharmacological advantages including enhanced biodistribution, targeted delivery, and improved safety profiles (15). Our rationally designed PDA@Fe/P leverages these properties while specifically targeting parasite iron metabolism (21, 22), with a dual mechanism of action: (i) inducing cytotoxic Fe^2+^ overload and oxidative stress-mediated parasite death; (ii) restoring ART sensitivity in resistant strains by supplementing the Fe^2+^ pool required for drug activation.

Despite these promising findings, several limitations must be addressed to advance PDA@Fe/P’s therapeutic potential. A key constraint is the transient nature of its *in vivo* efficacy: while it reduces *P. berghei* parasitemia (days 4–7), failure to extend host survival limits translational value, highlighting the need for strategies to prolong its bioactive window (e.g., optimized formulations or sustained-release delivery systems). Moreover, its relatively high IC_50_ value indicates that PDA@Fe/P is unlikely to serve as a standalone therapeutic agent, underscoring the need for structural modifications or combinatorial approaches to enhance potency. Importantly, caution is warranted, as multiple clinical and epidemiological reports have suggested that iron supplementation in malaria-endemic regions can increase the risk of severe malaria episodes and associated mortality. This potential hazard needs to be carefully considered when designing iron-based interventions, particularly in vulnerable populations. Furthermore, detailed pharmacokinetic properties—including absorption, distribution, metabolism, and excretion—remain uncharacterized; elucidating these parameters will be critical for guiding dosing regimens and safety assessments. While annexin V labeling represents another valuable assay to further evaluate the cytocompatibility of PDA@Fe/P, this experiment will be scheduled for future work due to time constraints. Addressing these limitations will be pivotal to advancing PDA@Fe/P as a viable and safe therapeutic agent in malaria management.

## MATERIALS AND METHODS

### Preparation of PDA@Fe/P complex

Dopamine hydrochloride (50 mg) was dissolved in 8 mL of Tris-HCl buffer (pH 8.8), followed by sequential addition of 1 mL of 100 mM FeCl_2_·4H_2_O solution and 1 mL of 20 mM sodium pyrophosphate solution under constant stirring. The reaction mixture was then incubated on a rotary shaker for 12 h to ensure uniform mixing. Upon completion, the product was isolated by centrifugation at 10,000 rpm for 10 min, subsequently washed to remove impurities, and finally dried to obtain the desired material.

### Characterization

Microstructural characterization was performed using a Hitachi HT7800 transmission electron microscope operated at 120 kV accelerating voltage to optimize contrast while minimizing beam damage. An X-ray diffractometer (XRD, Bruker D8 Advance) with Cu Kα (*λ* = 1.5406 Å) radiation generated at 40 mA and 40 kV was used to investigate the structure of the nanospheres in the 2*θ* range of 5°–90°. Elemental composition and chemical state analysis were performed using a Thermo Scientific ESCALAB 250Xi XPS system equipped with a monochromatic Al Kα X-ray source (1,486.6 eV). Survey scans (0–1,350 eV) were acquired at a pass energy of 100 eV with 1.0 eV step size to identify all detectable elements. High-resolution regional scans were subsequently collected for C 1s, N 1s, O 1s, P 2p, and Fe 2p core levels at a 20 eV pass energy with a 0.05 eV step size. The analysis area was approximately 500 μm in diameter with the electron take-off angle set at 90° relative to the sample surface.

### Release of Fe²^+^ from PDA@Fe/P

The release profile of Fe^2+^ from PDA@Fe/P was assessed in pH 7.4 PBS buffer at 37°C under static incubation. Briefly, PDA@Fe/P dispersions were prepared in PBS and maintained at 37°C. Aliquots of the supernatant were collected at predetermined time points (30 min, 1 h, 2 h, 4 h, 8 h, 16 h, and 24 h), and subsequently at every 24 h interval until the end of the study period.

At each time point, the supernatant was separated by centrifugation, and Fe^2+^ concentration was measured using a Ferric and Ferrous Ion Assay Kit (S1066S, Beyotime Biotechnology) following the manufacturer’s protocols. Absorbance was recorded at 593 nm with a microultraviolet spectrophotometer to quantify Fe^2+^ release.

### Parasite culture

All *P. falciparum* strains used in this study were maintained under standard *in vitro* culture conditions in our laboratory, including laboratory-adapted strains 3D7, 803, Dd2, and K1, as well as the field isolate SBC. The SBC isolate, obtained in 2013 from a Chinese worker returning from Equatorial Guinea (51), was CQsensitive, as was the laboratory strain 3D7, whereas K1, Dd2, and 803 exhibited CQ resistance. The K13 transgenic 3D7 Kelch13 C580Y strain (3D7 background) (52) was kindly provided by the Jiangsu Institute of Parasitic Diseases, Wuxi, China. Parasite cultures were maintained following established Trager-Jensen methods with specific modifications. The culture system utilized O-positive human erythrocytes (2% hematocrit) in complete medium (RPMI-1640 supplemented with 2 mg/mL NaHCO_3_, 50 μg/mL hypoxanthine, 5.96 mg/mL HEPES, 1% Albumax II, and 40 μg/mL gentamicin). For experimental procedures, cultures were standardized to 1% parasitemia and 2% hematocrit, and then incubated under controlled atmospheric conditions (5% O_2_, 5% CO_2_, and 90% N_2_) at 37°C with constant humidity.

To ensure stage-specific synchronization, parasites underwent two rounds of 5% sorbitol treatment at 40-h intervals targeting ring-stage forms. The synchronization protocol involved: (i) centrifugation of ring-stage cultures followed by washing with incomplete RPMI-1640 medium, (ii) 10-min incubation in 5% sorbitol at 37°C, and (iii) subsequent washing and resuspension in fresh medium. Parasite development was monitored through daily microscopic examination, with parasitemia quantification performed by analyzing Giemsa-stained thin blood smears (minimum 10,000 erythrocytes counted per sample). This rigorous synchronization protocol yielded greater than 95% ring-stage parasites for subsequent experiments, as confirmed by morphological assessment.

### Assessment of *in vitro* antimalarial activity of iron-based nanomaterials

The antimalarial activity of iron-based nanomaterials was evaluated against highly synchronized ring-stage parasites using a standardized 72-h growth inhibition assay. For preliminary screening, synchronized parasite cultures (200 μL/well at 1% parasitemia and 2% hematocrit) were exposed to 100 μg/mL initial concentrations of PDA@Fe/P, PDA@Fe(III)P, Fe-C, and Fe-N in 96-well plates, with subsequent twofold serial dilutions. Comprehensive dose-response analyses were performed for PDA@Fe/P and its precursor FeCl_2_·4H_2_O across six concentrations (PDA@Fe/P: 25–800 μg/mL and FeCl_2_·4H_2_O: 472–15,090 μg/mL). Following 72-h incubation under standard culture conditions (37°C, 5% CO_2_, and 5% O_2_), parasite viability was quantified through microscopic examination of Giemsa-stained thin blood smears, with parasitemia determined by counting at least 10,000 erythrocytes per sample. The half-maximal inhibitory concentrations (IC_50_) were calculated using nonlinear regression analysis in GraphPad Prism 8.0, with each experimental condition evaluated with two independent biological replicates containing three technical replicates each. Control wells containing equivalent concentrations of PBS in complete medium were included for baseline comparison.

### Hemolytic activity

Defibrinated rabbit blood was washed three times with phosphate-buffered saline (PBS) followed by centrifugation at 1,600 rpm for 5 min. The washed RBCs were then resuspended in PBS to achieve a 2% hematocrit, with the suspension containing either test compounds (PDA@Fe/P, FeCl_2_·4H_2_O, or CQ), negative control (PBS), or positive control (0.4% Triton X-100). After incubation at 37°C for 30 min, the plates were centrifuged at 1,600 rpm for 5 min, and the absorbance of supernatants was measured at 543 nm. The positive control samples treated with Triton X-100 were diluted 10-fold prior to absorbance measurement. The percentage of hemolysis was calculated using the following formula: Hemolysis (%) = [(*A*_sample_ – *A*_PBS_) / (*A*_Triton X-100_ – *A*_PBS_)] × 100%, where *A*_sample_, *A*_PBS_, and *A*_Triton X-100_ represent the absorbance values of the test compound, PBS control, and Triton X-100 control, respectively.

### Cytotoxicity

The HUVECs were maintained under standard culture conditions. For cell revival, frozen vials were rapidly thawed in a 37°C water bath, transferred to serum-free DMEM medium, and centrifuged (1,500 rpm, 5 min). Cell pellets were resuspended in complete DMEM medium (supplemented with 10% FBS) and cultured in T25 flasks at 37°C with 5% CO_2_. Upon reaching 70–80% confluence, cells were passaged using 0.25% trypsin-EDTA digestion, washed with serum-free DMEM, and reseeded at appropriate densities.

Cell viability was assessed using CCK-8 assays. Briefly, HUVECs (5 × 10^3^ cells/well in 100 μL complete medium) were seeded in 96-well plates and incubated for 24 h. Test compounds were then added at specified concentrations. After 48 h incubation, cells were washed and incubated with CCK-8 solution (10% vol/vol in DMEM) for 2 h. Absorbance was measured at 450 nm using a microplate reader. Data analysis was performed using GraphPad Prism 8.0, with statistical significance set at *P* < 0.05.

### Four-day suppressive test

The *in vivo* antimalarial effect of PDA@Fe/P was evaluated using a standard 4-day suppressive test in female BALB/c mice (6–8 weeks old, weighing 18–22 g), maintained under specific pathogen-free (SPF ) conditions with *ad libitum* access to food and water. The *P. berghei* ANKA murine malaria model was established by intraperitoneal inoculation of 1 × 10⁷ parasitized erythrocytes (in 100 μL saline) per mouse, following parasite reactivation from −80°C cryopreservation. Infection progression was monitored through daily Giemsa-stained thin blood smears of tail-vein blood, with treatment initiated when parasitemia reached approximately 20%. For chemotherapeutic evaluation, the standardized Peters' 4-day suppressive test was employed, commencing 3 h post-infection (designated Day 0). Experimental groups (*n* = 6 mice/group) received daily intravenous treatments for four consecutive days (Days 0–3): PDA@Fe/P (1.4, 4.2, and 12.6 mg/kg), CQ (78.16 μmol/kg as positive control), or PBS (negative control). Parasitemia quantification and survival monitoring were performed from Day 4 onward through microscopic examination of methanol-fixed, Giemsa-stained blood smears, with at least 10,000 erythrocytes counted per sample to determine infection rates.

### *In vitro* drug assays

RSA^0–3h^ was conducted as previously described (53). Briefly, late-stage schizonts were purified from synchronous *P. falciparum* cultures containing only late-stage schizonts and newly invaded rings, adjusted to 2% hematocrit and 1% parasitemia by adding uninfected erythrocytes, and dispensed (2 mL per well in a 24-well culture plate) into two parallel cultures. Parasite cultures were distributed in 1 mL aliquots into 24-well plates and subjected to pharmacological treatments under controlled conditions (37°C, 5% O_2_, and 5% CO_2_). Parasite cultures were treated: (i) 700 nM DHA, (ii) 100, 50, and 25 μg/mL PDA@Fe/P, (iii) combination therapy (DHA + PDA@Fe/P), and (iv) DMSO vehicle control (with equivalent solvent concentrations across all samples).

Parasitemia was assessed by flow cytometry using SYBR Green I (Invitrogen)/ MitoTracker Deep Red FM (Invitrogen) and Giemsa-stained blood smears. Parasite-infected erythrocytes were quantitatively analyzed using a dual-fluorescence flow cytometry protocol. Cell suspensions were stained with 0.3 μM each of Mito and SYBR Green I in PBS (pH 7.4) at 37°C for 30 min, followed by two washes with PBS to remove unbound dye. Samples were analyzed on an ACSCalibur flow cytometer (BD Biosciences) with the following optical configuration: SYBR Green I detection at 488 nm excitation/525 nm emission (50 nm bandwidth), and MITO detection at 561 nm excitation/695 nm emission (50 nm bandwidth). Data analysis was performed using FlowJo software (version 10, FlowJo LLC) employing a sequential gating strategy: (i) exclusion of cellular debris and doublets based on forward and side scatter properties, followed by (ii) selection of SYBR Green I+ dual-positive populations representing intact, parasite-infected erythrocytes. Median fluorescence intensity values were calculated for both fluorescence channels to quantify parasite load and viability.

### Assay for GSH/GSSG and MDA

Cortical GSH content was measured using a GSH and GSSG assay kit (#S0053) as per the manufacturer’s instructions (Beyotime Biotechnology, Shanghai, China). The content was measured using a colorimetric microplate reader (Thermo Scientific Multiskan FC, OD = 593 nm). GSH content was expressed as μmol/g, and the GSH content of the test samples was calculated as follows: Total Glutathione – GSSG × 2. MDA, the final product of lipid peroxidation, reacts with thiobarbituric acid to form a red product with maximum absorption at 532 nm. To detect the extent of lipid peroxidation in parasites, PDA@Fe/P of different concentrations was incubated with parasites for 24 h at 37°C, and then the cells were used to measure MDA content using a micro-MDA assay reagent kit (KeyGEN BioTECH, Jiangsu, China).

### Statistical analysis

GraphPad Prism software was used to perform a two-tailed Student’s *t*-test on unpaired samples for comparisons between experimental groups. For all tests, a *P* value was considered significant if it was <0.05 (*), <0.01 (**), or <0.001 (***).

## SUPPLEMENTAL MATERIAL

Supplemental material aac.01390-25-s0001.docx Fig. S1 to S3; Tables S1 and S2.

ASM does not own the copyrights to Supplemental Material that may be linked to, or accessed through, an article. The authors have granted ASM a non-exclusive, world-wide license to publish the Supplemental Material files. Please contact the corresponding author directly for reuse.
