# Supplementary material for: A Fe-incorporated bioreactor platform exhibiting antimalarial activity and enhanced response to artemisinin
Source: Antimicrob Agents Chemother. 2026 Feb 20;70(4):e01390-25. doi: 10.1128/aac.01390-25 (PMC13041302; doi:10.1128/aac.01390-25)
Supplement: Supplemental material — Fig. S1 to S3; Tables S1 and S2. [file aac.01390-25-s0001.docx]

**Supporting Figures**

**
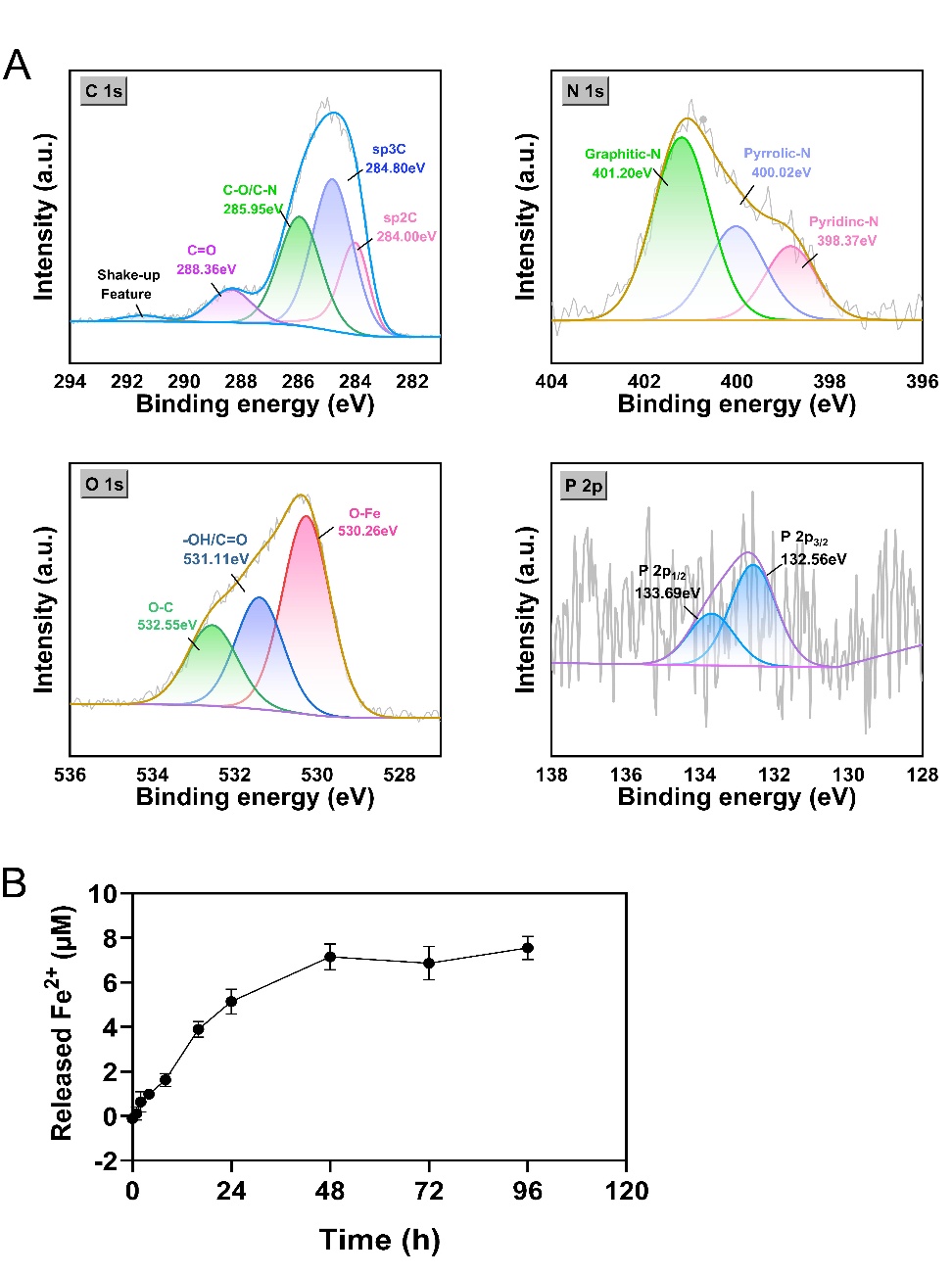
**

**Supplementary Fig. 1** (A) Synthesis and characterization of PDA@Fe/P include high-resolution C 1s, N 1s, O 1s, and P 2p peaks. (B) Fe^2+^ release profile of PDA@Fe/P in culture medium at 37°C showing sustained liberation of iron ions over time. Fe^2+^ concentrations were measured using a probe-based assay.


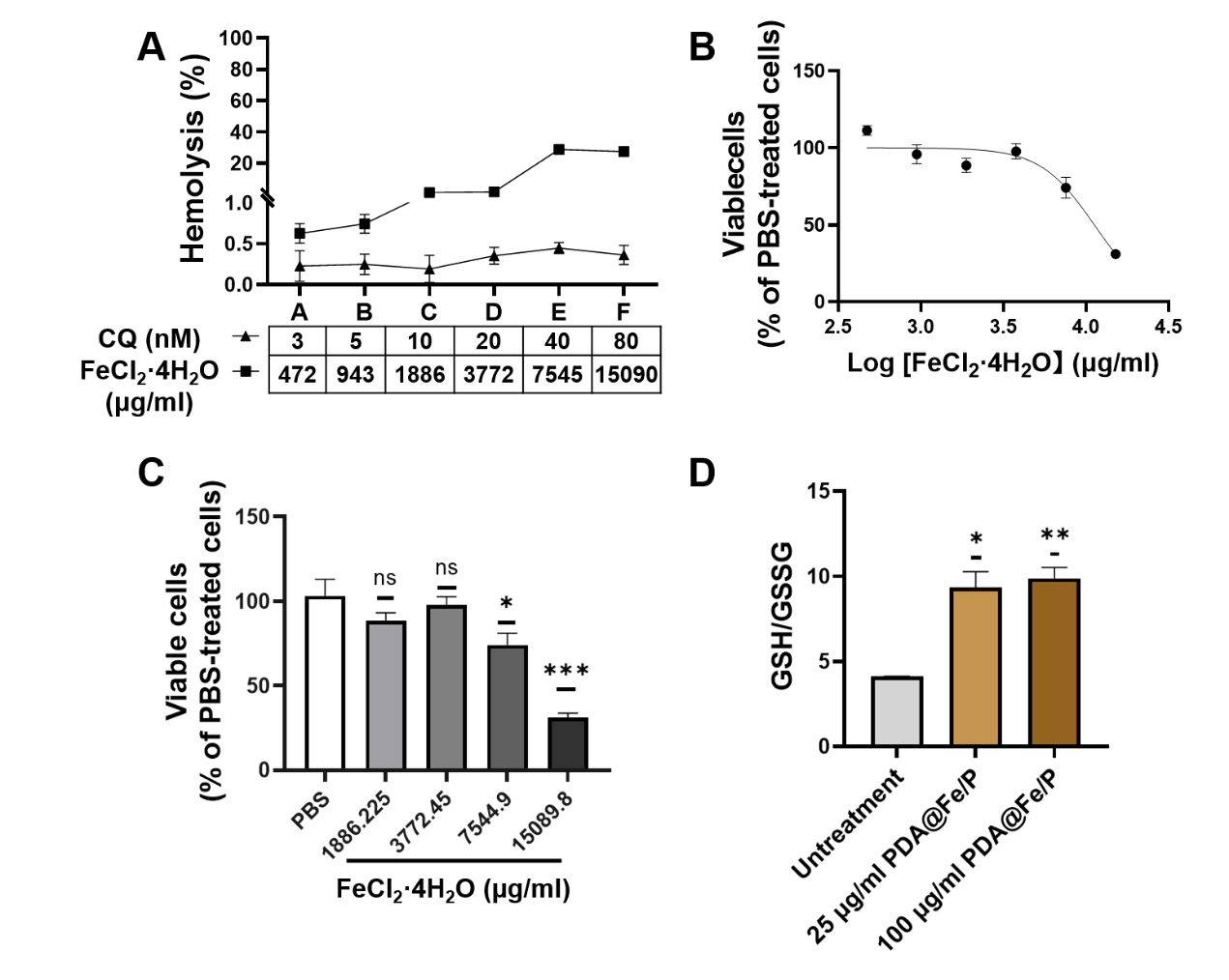
**Supplementary Fig. 2** (A) Hemolysis assessment of FeCl_2_·4H_2_O and CQ (n=3). (B) and (C) Cytotoxicity of different concentrations of FeCl_2_·4H_2_O to HUVECs. (D) Effect of PDA@Fe/P on GSH/GSSG ratio in red blood cells (RBCs).

**
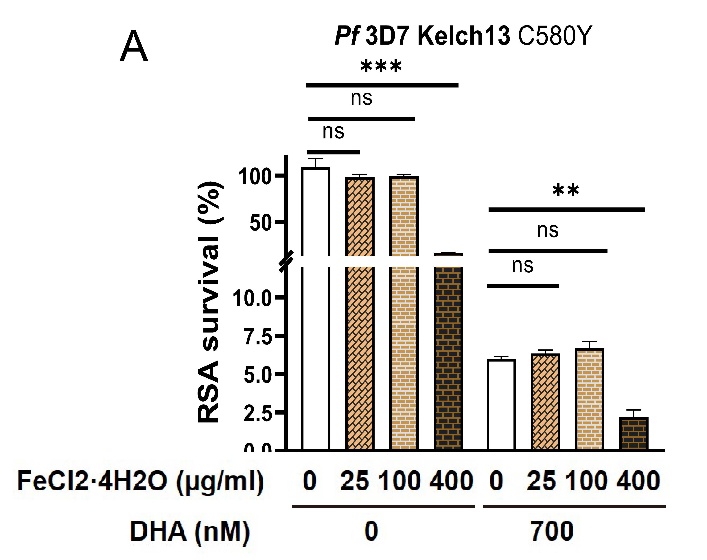
**

**Supplementary Fig. 3** (A) RSA results showing that FeCl_2_ synergizes with DHA in 3D7 Kelch13 C580Y, with notable toxicity at high concentrations. FeCl_2_ alone displays measurable antimalarial activity. Values are mean ± SD (n = 3).

**Supporting Tables**

**Table S1.** Antimalarial efficacy of different nanomaterials against *Plasmodium* parasites.

| **Concentration**  **（μg/ml）** | **Inhibition (%) of iron-based antimalarial nanomaterials** | | | | |
| --- | --- | --- | --- | --- | --- |
|  | **PDA@Fe/P** | **PDA@Fe(Ⅲ)P** | **Fe-C** | **Fe-N** | **PDA** |
| 100.0 ^a^ | 60.0±0.23 | 43.1±0.49 | 3.6±0.36 | 7.0±1.39 | 18.5±2.22 |
| 50.0 ^a^ | 43.2±0.76 | 32.2±0.64 | 6.3±0.24 | 4.2±0.76 | 14.6±2.24 |
| 25.0 ^a^ | 18.8±0.50 | 10.7±0.87 | 7.3±0.97 | 8.8±0.35 | 20.1±3.72 |
| 12.5 | 5.4±1.32 | 2.4±0.39 | 0.6±1.06 | 1.0±0.96 | 20.2±3.80 |
| 6.3 | 3.0±1.09 | 3.1±1.74 | 1.0±1.21 | 0.2±0.76 | 2.4±2.94 |
| 3.1 | 0.6±0.44 | 0.5±0.44 | 0.4±1.35 | 0±0.99 | 0.7±3.86 |
| 0 | 0 | 0 | 0 | 0 | 0 |

^a^ Statistically significant difference between PDA@Fe/P and all other groups at the same concentration (*P < 0.0001*).

**Table S2.**  IC_50_ values of CQ, PDA@Fe/P, and FeCl_2_·4H_2_O against *P. falciparum*strains (3D7, 803, Dd2, and K1) and a field isolate (SBC).

| ***P. f* strains** | **IC_50_ (95% Cl) of iron-based antimalarial drug** | | |
| --- | --- | --- | --- |
|  | **PDA@Fe/P【µg/ml】** | **FeCl_2_·4H_2_O【µg/ml】** | **CQ 【nM】** |
| 3D7 | 129.1（104.8-158.3） | 2330（2051-2648） | 21.08（20.21-22.21） |
| SBC | 168.8（153.7-185.2） | 3995（3307-4840） | 22.11（20.25-24.70） |
| K1 | 209.3（175.8-248.2） | 4668（4092-5322） | 276.4（253.2-301.7） |
| Dd2 | 198.6（175.9-224.0） | 3798（3210-4498） | 297.3（277.3-318.8） |
| 803 | 168.4（142.3-198.6） | 4015（3530-4576） | 337.3（286.6-390.5） |
